# Supplementary material for: Assessing Knowledge and Attitude of Healthcare Professionals on Biosimilars: A National Survey for Pharmacists and Physicians in Taiwan
Source: Healthcare (Basel). 2021 Nov 21;9(11):1600. doi: 10.3390/healthcare9111600 (PMC8619957; doi:10.3390/healthcare9111600)
Supplement: Supplementary file 1 [file healthcare-09-01600-s001.zip › healthcare-1433565-supplementary.pdf]

Supplementary Material

Table S1. The association between attitude statements and demographic characteristics.

Statements	**Q10.** I possess a good understanding of biosimilar products.				**Q11.** Generally, I feel comfortable prescribing biosimilars to patients because I am confident about their safety and efficacy.				**Q12.** The nonproprietary name of biosimilars should be distinguishable from the reference product.				**Q13.** If necessary, I accept the switch to a biosimilar product for patients receiving treatment of its reference product.			
	**D**	**N**	**A**	***p-value***	**D**	**N**	**A**	***p-value***	**D**	**N**	**A**	***p-value***	**D**	**N**	**A**	***p-value***
	n (%)				n (%)				n (%)				n (%)			
**Specialty**				0.001*				0.10				0.20				0.02*
Pharmacist	38 (23.2)	39 (23.8)	87 (53.0)		37 (22.6)	44 (26.8)	83 (50.6)		16 (9.8)	8 (4.9)	139 (85.3)		39 (23.9)	38 (23.3)	86 (52.8)	
Oncology	15 (12.2)	28 (22.8)	80 (65.0)		35 (28.7)	22 (18.0)	65 (53.3)		7 (5.7)	7 (5.7)	109 (88.6)		42 (34.1)	23 (18.7)	58 (47.2)	
Rheumatologist	8 (7.4)	19 (17.6)	81 (75.0)		38 (35.2)	19 (17.6)	51 (47.2)		3 (2.8)	8 (7.4)	97 (89.8)		40 (37.4)	30 (28.0)	37 (34.6)	
**Gender**				0.007*				0.37				0.96				0.97
Male	31 (12.6)	45 (18.2)	171 (69.2)		67 (27.2)	47 (19.1)	132 (53.7)		16 (6.5)	14 (5.7)	216 (87.8)		77 (31.3)	56 (22.8)	113 (45.9)	
Female	28 (19.9)	38 (27.0)	75 (53.2)		41 (29.1)	34 (24.1)	66 (46.8)		9 (6.4)	9 (6.4)	123 (87.2)		43 (30.7)	31 (22.1)	66 (47.1)	
**Age group**				0.96				0.77				0.97				0.66
20-39	22 (24.4)	19 (21.1)	49 (54.4)		24 (26.7)	25 (27.8)	41 (45.6)		5 (5.6)	6 (6.7)	79 (87.8)		25 (27.8)	19 (21.1)	46 (51.1)	
40-49	22 (12.6)	42 (24.0)	111 (63.4)		51 (29.1)	35 (20.0)	89 (50.9)		12 (6.9)	8 (4.6)	154 (88.5)		54 (31.2)	45 (26.0)	74 (42.8)	
50-59	14 (15.1)	15 (16.1)	64 (68.8)		25 (27.2)	19 (20.7)	48 (52.2)		7 (7.5)	6 (6.5)	80 (86.0)		28 (30.1)	18 (19.4)	47 (50.5)	
60+	3 (8.1)	10 (27.0)	24 (64.9)		10 (27.0)	6 (16.2)	21 (56.8)		2 (5.4)	3 (8.1)	32 (86.5)		14 (37.8)	9 (24.3)	14 (37.8)	
**Hospital types**				0.04*				0.66				0.007*				0.43
Medical	17 (11.8)	25 (17.4)	102 (70.8)		43 (30.1)	32 (22.4)	68 (47.6)		4 (2.8)	4 (2.8)	136 (94.4)		44 (30.8)	38 (26.6)	61 (42.7)	
Regional	44 (17.5)	61 (24.3)	146 (58.2)		67 (26.7)	53 (21.1)	131 (52.2)		22 (8.8)	19 (7.6)	209 (83.6)		77 (30.8)	53 (21.2)	120 (48.0)	
**Regions**				0.34				0.65								
North	32 (17.4)	41 (22.3)	111 (60.3)		55 (29.9)	37 (20.1)	92 (50.0)		13 (7.1)	8 (4.3)	163 (88.6)	0.13	60 (32.8)	40 (21.9)	83 (45.4)	0.51
Central	17 (17.9)	20 (21.1)	58 (61.1)		21 (22.1)	23 (24.2)	51 (53.7)		2 (2.1)	8 (8.5)	84 (89.4)		23 (24.2)	24 (25.3)	48 (50.5)	
South	9 (9.2)	20 (20.4)	69 (70.4)		28 (28.9)	23 (23.7)	46 (47.4		10 (10.2)	7 (7.1)	81 (82.7)		31 (32.0)	26 (26.8)	40 (41.2)	

Statements	**Q14.** I would only prescribe a biosimilar product to those who have never received treatment with its reference drug (i.e., biologic-naïve).				**Q15.** I am familiar with the regulations of the Taiwan Food and Drug Administration (TFDA) on biosimilars.				**Q16.** The non-proprietary name of biosimilar drugs should not be distinguishable from the reference product (respondent reliability).			
	**D**	**N**	**A**	***p-value***	**D**	**N**	**A**	***p-value***	**D**	**N**	**A**	***p-value***
	n (%)				n (%)				n (%)			
**Specialty**				0.21				0.91				0.002*
Pharmacist	58 (35.4)	50 (30.5)	56 (34.1)		54 (32.9)	43 (26.2)	67 (40.9)		132 (80.5)	11 (6.7)	21 (12.8)	
Oncology	50 (40.7)	38 (30.9)	35 (28.5)		42 (34.1)	29 (23.6)	52 (42.3)		108 (87.8)	11 (8.9)	4 (3.3)	
Rheumatologist	40 (37.0)	23 (21.3)	45 (41.7)		32 (29.6)	31 (28.7)	45 (41.7)		99 (91.7)	7 (6.5)	2 (1.9)	
**Gender**				0.15				0.55				0.42
Male	96 (38.9)	60 (24.3)	91 (36.8)		77 (32.2)	62 (25.1)	108 (43.7)		211 (85.4)	21 (8.5)	15 (6.1)	
Female	50 (35.5)	47 (33.3)	44 (31.2)		50 (35.5)	37 (26.2)	54 (38.3)		121 (85.7)	8 (5.7)	12 (8.5)	
**Age group**				0.57				0.19				0.01*
20-39	35 (38.9)	29 (32.2)	26 (28.9)		36 (40.0)	26 (28.9)	28 (31.1)		77 (85.6)	5 (5.6)	8 (8.9)	
40-49	58 (33.1)	50 (28.6)	67 (38.3)		52 (29.7)	47 (26.9)	76 (43.4)		155 (88.6)	8 (4.6)	12 (6.9)	
50-59	39 (41.9)	22 (23.7)	32 (34.4)		26 (28.0)	25 (26.9)	42 (45.2)		78 (83.9)	8 (8.6)	7 (7.5)	
60+	16 (43.2)	10 (27.0)	11 (29.7)		14 (37.8)	5 (13.5)	18 (4836)		29 (78.4)	8 (21.6)	0 (0)	
**Hospital types**				0.57				0.66				0.008*
Medical	51 (35.4)	45 (31.3)	48 (33.3)		45 (31.3)	35 (24.3)	64 (44.4)		133 (92.4)	8 (5.6)	3 (2.1)	
Regional	97 (38.6)	66 (26.3)	88 (35.1)		83 (33.1)	68 (27.1)	100 (39.8)		206 (82.1)	21 (8.4)	24 (9.6)	
**Regions**												
North	75 (40.8)	53 (28.8)	56 (30.4)	0.05	61 (33.2)	51 (27.7)	72 (39.1)	0.20	157 (85.3)	16 (8.7)	11 (6.0)	0.70
Central	33 (34.7)	32 (33.7)	30 (31.6)		31 (32.6)	17 (17.9)	47 (49.5)		84 (88.4)	6 (6.3)	5 (5.3)	
South	29 (29.6)	23 (23.5)	46 (46.9)		28 (28.6)	31 (31.6)	39 (39.8)		83 (84.7)	6 (6.1)	9 (9.2)	

Note: The attitude question statements were represented with a 5-point Likert data, D: Strongly disagree and disagree, N: Neutral, A: Strongly agree and agree; Gender [n = 388, P (160), O (121), R (107)], excluded the "prefer not to say" category; Regions [n = 377, P (156), O (116), R (105)], excluded the "Eastern region" category. *$P < 0.05$.
